# Complete mitochondrial genome of the Woodland Brown, *Lopinga achine* Scopoli, 1763 (Nymphalidae: Satyrinae) and its phylogenetic analysis

**DOI:** 10.1080/23802359.2022.2070042

**Published:** 2022-05-03

**Authors:** Jia-Ling Wu, Ting-Ting Bao, Gang Sun, Ying Xiao, Yan Fang, Qing-Hui Shi

**Affiliations:** a Medical Plant Exploitation and Utilization Engineering Research Center, Sanming University, Sanming, P. R. China; b Fujian Provincial Key Laboratory of Resources and Environment Monitoring & Sustainable Management and Utilization, Sanming University, Sanming, P. R. China

**Keywords:** Satyrinae, *Lopinga achine*, mitochondrial genome, phylogeny

## Abstract

In this study, the complete mitochondrial genome (mitogenome) of the Woodland Brown, *Lopinga achine* Scopoli, 1763 (Nymphalidae: Satyrinae) was determined to be 15,284 bp in size, including 37 typical mitochondrial genes and a control region. The gene content and arrangement of the mitogenome are identical to that of the majority of other sequenced nymphalids. All protein-coding genes (PCGs) are started with the conventional ATN codons, except for *cox1* gene which is initiated by atypical CGA(R) codon. Nine PCGs use a typical stop codon of TAA, whereas the remaining PCGs (*cox1*, *cox2*, *nad4*, *nad5*) end with an incomplete T. The length of *rrnL* and *rrnS* are 1333 and 755 bp, respectively, separated by *trnV*. The phylogenetic tree inferred with Bayesian inference method reveals the phylogenetic relationships among the four tribes of Satyrinae analyzed as ((Satyrini + Melanitini) + (Elymniini + Amathusiini)). The newly sequenced species *L. achine* was clustered together with other two species of Parargina and formed a sister group with two species of the genus *Lethe* within Satyrini.

The subfamily Satyrinae is one of the most diverse butterfly groups of Nymphalidae with about 2,500 species distributed on all continents except Antarctica (Ackery et al. 1999; Peña and Wahlberg 2008). Owing to its high diversity, many Satyrinae species have been intensely investigated as model organisms in various research fields such as ecology (Schmitt and Haubrich 2008), developmental biology (Oliver et al. 2012), and conservation biology (Slamova et al. 2013). Regarding the classifications of Satyrinae, nine tribes and 16 subtribes have been defined (Marín et al. 2011). However, phylogeny among them remains largely unresolved (Yang and Zhang 2015; Yang et al. 2020; Sun et al. 2021). In recent decades, the insect mitochondrial genomes (mitogenomes) are being increasingly employed to explore the evolution and phylogenetic relationships in diverse insect taxa (Cameron 2014; Wu et al. 2014; Yang et al. 2020). Therefore, the complete mitogenome of the Woodland Brown, *Lopinga achine* Scopoli, 1763 (Nymphalidae: Satyrinae: Satyrini) was determined and analyzed in this study, which would be helpful to make the subdivision of Satyrinae clearer.

A specimen of *L. achine* was collected by Qinghui Shi from Minshan Mountain, Minxian County, Dingxi City, Gansu Province, China (34.433 N, 104.417 E) in June 2015. The fresh individual was preserved in absolute ethyl alcohol and deposited at the Medical Plant Exploitation and Utilization Engineering Research Center, Sanming University (Qinghui Shi, 214213898@qq.com) under the Voucher number SMU-20150627. Total genomic DNA was extracted and purified from thorax muscle of *L. achine* using the Rapid Animal Genomic DNA Isolation Kit (Sangon Biotech, Shanghai, China). One library (410 bp) was constructed, and an Illumina HiSeq platform was used for sequencing with the strategy of paired-end (Majorbio, Shanghai, China). Approximately 20,582,496 raw reads were obtained from *L. achin* mitogenome. Adapter sequences, low quality reads, reads with >10% of unknown bases, and ambiguous bases were removed to obtain high quality assembly. About 18,852,758 clean reads were gained and assembled by SOAPdenovo2 (Luo et al. 2012). The assembled mitogenome was annotated using the online DOGMA tool (http://dogma.ccbb.utexas.edu/) and further corrected manually. This study including the collection of sample was approved by the Scientific Research Administrative of Sanming University and complied with national and local legislation.

The complete mitogenome of *L. achine* is 15,284 bp in length (GenBank accession no. MT117843), consisting of 13 protein-coding genes (PCGs), 22 transfer RNA (tRNA) genes, 2 ribosomal RNA (rRNA) genes, and a control region. The gene content and arrangement of *L. achine* mitogenome are identical to other published Satyrinae mitogenomes (Chen et al. 2020; Sun et al. 2021). The overall GC content of the mitogenome is 20.5%, as the percentages of A, T, C, and G are 38.9%, 40.5%, 12.9%, and 7.6%, respectively. Additionally, 10 intergenic spacers (96 bp in total) and 13 overlapping regions (33 bp in total) are scattered throughout the whole mitogenome. Besides the control region, the largest noncoding region (60 bp in length) was located between gene *trnQ* and *nad2*. All PCGs are initiated by the typical codon of ATN, except for *cox1* gene, which starts with the unusual CGA as found in most other determined Satyrinae mitogenomes (Shi et al. 2019; Yang et al. 2020). Nine PCGs use TAA as the termination codons, while four PCGs (*cox1*, *cox2*, *nad5*, *nad4*) stop with an incomplete T. The *rrnL* (1333 bp) and *rrnS* (755 bp) are separated by *trnV*.

To better understanding the phylogeny of Satyrinae, the phylogenetic tree was reconstructed based on concatenated nucleotide sequences of 13 PCGs and 2 rRNAs of *L. achine* and other 23 representative mitogenomes from Satyrinae and two outgroup species from Calinaginae and Charaxinae (Figure 1). The 13 PCGs and 2 rRNAs were first aligned individually using MEGA7.0 software (Kumar et al. 2016), then concatenated using DAMBE7 (Xia 2018). The best model (GTR + I+ G) for concatenate sequences under the corrected Akaike Information Criterion using jModeltest 2.1.10 (Darriba et al. 2012) was selected. The Bayesian inference (BI) analysis was performed using MrBayes 3.2 (Ronquist et al. 2012), then four simultaneous Markov chains ran for 2 million generations and trees were sampled every 100 generations, with a burn-in of 25%. The BI analysis recovers the phylogenetic relationships among the four tribes of Satyrinae analyzed as ((Satyrini + Melanitini) + (Elymniini + Amathusiini)), which is identical to that of Yang et al. (2020), but showing difference with that of other studies (Yang and Zhang 2015; Sun et al. 2021). The newly sequenced species *L. achine* clustered together with other two species of Parargina and formed a sister group with two species of the genus *Lethe* within Satyrini. Nevertheless, it should be noted that only eight of the 13 subtribes of Satyrini are analyzed in this study, and further effort is needed to improve the understanding of the whole Satyrini phylogeny based on mitogenome sequences of increased sampling.

**Figure 1.**
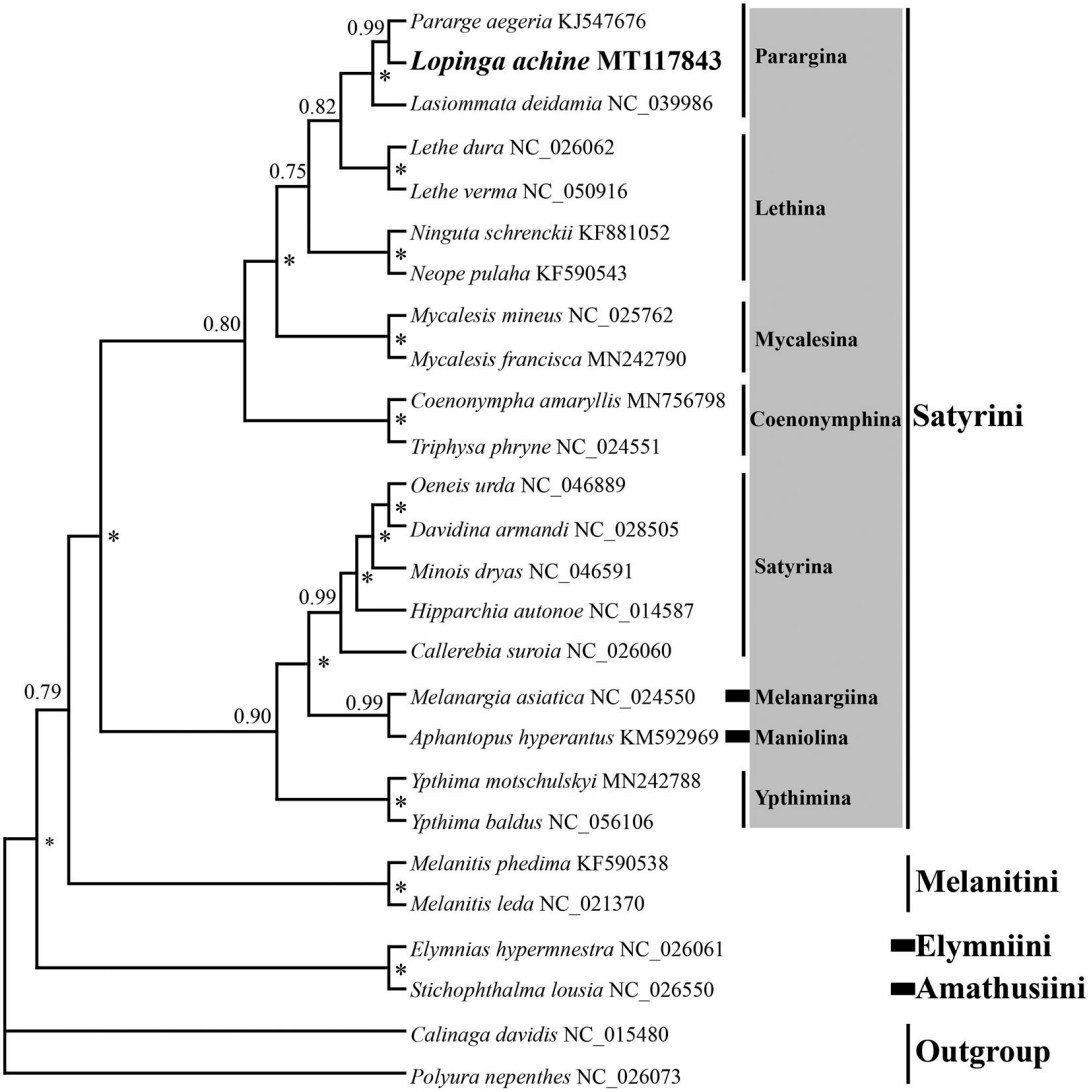
The Bayesian inference (BI) phylogenetic tree of *Lopinga achine* and other Nymphalidae butterflies. Phylogenetic reconstruction was done from a concatenated matrix of 13 protein-coding mitochondrial genes and 2 ribosomal RNA genes. The numbers beside the nodes correspond to the posterior probability values (* = 1.00). Alphanumeric terms indicate the GenBank accession numbers.

## Data Availability

The genome sequence data that support the findings of this study are openly available in GenBank of NCBI at https://www.ncbi.nlm.nih.gov, under the accession no. MT117843. The associated BioProject, SRA, and Bio-Sample numbers are PRJNA795845, SRR17761215, and SAMN24783966, respectively.
